# Looking at the positives: proactive management of STIs in people with HIV

**DOI:** 10.1186/s12981-018-0216-9

**Published:** 2018-12-21

**Authors:** Carole Khaw, Daniel Richardson, Gail Matthews, Tim Read

**Affiliations:** 1 Adelaide Sexual Health Centre (Clinic 275), Infectious Diseases Unit, Royal Adelaide Hospital, 275 North Terrace, Adelaide, SA 5000 Australia; 2 School of Medicine, University of Adelaide, Adelaide, SA Australia; 3 Department of Sexual Health and HIV Medicine, Brighton and Sussex Medical School, Brighton, UK; 4 Department of Sexual Health and HIV Medicine, Brighton and Sussex University NHS Trust, Brighton, UK; 5 The Kirby Institute, University of New South Wales, Sydney, NSW Australia; 6 Melbourne Sexual Health Centre, Alfred Health, Melbourne, VIC Australia; 7 Central Clinical School, Monash University, Melbourne, VIC Australia

**Keywords:** HIV, Co-infection, HCV, Hepatitis C, Syphilis, Antimicrobial resistance, Gonorrhoea, Proctitis

## Abstract

Patients who are HIV-positive and co-infected with other sexually transmitted infections (STIs) are at risk of increased morbidity and mortality. This is of clinical significance. There has been a dramatic increase in the incidence of STIs, particularly syphilis, gonorrhoea, *Mycoplasma genitalium* and hepatitis C virus (HCV) in HIV-positive patients. The reasons for this are multifactorial, but contributing factors may include effective treatment for HIV, increased STI testing, use of HIV pre-exposure prophylaxis and use of social media to meet sexual partners. The rate of syphilis–HIV co-infection is increasing, with a corresponding increase in its incidence in the wider community. HIV-positive patients infected with syphilis are more likely to have neurological invasion, causing syndromes of neurosyphilis and ocular syphilis. HIV infection accelerates HCV disease progression in co-infected patients, and liver disease is a leading cause of non-AIDS-related mortality among patients who are HIV-positive. Since several direct-acting antivirals have become subsidised in Australia, there has been an increase in treatment uptake and a decrease in HCV viraemia in HIV-positive patients. The incidence of other sexually transmitted bacterial infections such as *Neisseria gonorrhoeae* and *M. genitalium* is increasing in HIV patients, causing urethritis, proctitis and other syndromes. Increasing antimicrobial resistance has also become a major concern, making treatment of these infections challenging. Increased appropriate testing and vigilant management of these STIs with data acquisition on antimicrobial sensitivities and antimicrobial stewardship are essential to prevent ongoing epidemics and emergence of resistance. Although efforts to prevent, treat and reduce epidemics of STIs in patients living with HIV are underway, further advances are needed to reduce the significant morbidity associated with co-infection in this patient setting.

## Background

In this paper, we summarise the presentations from the 2017 HIV Innovation Forum in Australia on the theme of “Proactive Management of STIs in People Living with HIV” The three presentations given under this theme were ‘Syphilis Co-infection In Patients who are HIV positive’, ‘Elimination HCV and HIV Co-infection In Australia’ and ‘Proctitis and Antimicrobial Resistance in the HIV clinic’. It should be emphasised that our objective in translating the key messages of these presentation’s into this report was not to offer a comprehensive systematic review of the topics, but to communicate, educate and summarise the useful overviews and practical clinical advice offered by all invited speakers. The report is therefore deliberately succinct. We hope that this format makes the information conveyed accessible to busy clinicians.

We have seen epidemics of sexually transmitted infections (STI), including syphilis, gonorrhoea, *Mycoplasma genitalium* and hepatitis C virus (HCV), in HIV-infected patients. The emergence of antimicrobial resistance has compounded some of these epidemics. Understanding how to manage sexually transmitted co-infections in people living with HIV is vital for reducing morbidity and mortality in this patient population and combating these epidemics.

## Syphilis co-infection in patients who are HIV-positive

Syphilis is a STI caused by the pathogenic spirochaete *Treponema pallidum* subsp. *pallidum*. The spirochaete varies from 6 to 15 μm in length and is 0.2 μm in width. With a doubling time of 30 to 50 h, *T. pallidum* is very difficult to culture in vitro [1]. Closely-related pathogenic treponemes cause endemic syphilis syndromes, such as bejel, yaws and pinta.

### History, diagnosis and treatment of syphilis

Bony remains from archaeological digs suggestive of syphilitic osteitis have been found in Europe and these pre-date the widely accepted timing of syphilis introduction to the continent (circa 1492) by approximately 100 years [2]. However, it can be difficult to distinguish whether these were a consequence of other treponemal infections. The spread of syphilis in Europe was rapid between 1492 and 1493, following the discovery of the Americas, with Christopher Columbus creating trade routes between the Americas and Europe, and the invasion of Naples by King Charles of France and his 50,000 soldiers.

Historically, syphilis research has been shrouded in controversy, as evidenced by the Oslo [3], Tuskagee [4] and Guatemala [5] experiments. The natural history of untreated syphilis in immunocompetent individuals is understood following human inoculation [6] and observational studies [7], with clearly defined stages and characteristic manifestations.

Laboratory testing is an important aspect of syphilis diagnosis and management. Tests can be categorised as direct detection, treponemal tests and non-treponemal tests (Table 1) [8].

**Table 1:** Diagnostic tests for syphilis

Approach	Method	Use	Features
Direct detection	Dark ground microscopy PCR		Require exudates and fluids from lesions PCR improving diagnostic thresholds
Treponemal tests	EIA TPHA/TPPA Western blot (IgG or IgM)	Screening	Highly sensitive Do not correlate with disease activity
Non-treponemal tests (against anti-cardiolipin antibodies)	VDRL RPR	Monitor treatment response Qualitative titre	Correlate with disease activity and treatment response Risk of false positive results

*EIA* enzyme immunoassay, *IgG* immunoglobulin G, *IgM* immunoglobulin M, *PCR* polymerase chain reaction, *TPHA Treponema pallidum* haemagglutination assay, *TPPA Treponema pallidum* particle agglutination assay, *RPR* rapid plasma reagin, *VDRL* venereal disease research laboratory

Historical treatments for syphilis included heat treatment, mercury treatment and salvarsan (arsenic) treatment. Currently, the preferred treatment for syphilis is penicillin G. Cerebrospinal fluid (CSF) studies have shown that standard benzathine penicillin (penicillin G) does not yield good CSF concentrations. However, this does not correlate with treatment failure [9]. Oral doxycycline is as effective as parenteral penicillin in the treatment of early syphilis [9–12]. Most international guidelines suggest benzathine penicillin for early syphilis, unless there is evidence of neurological disease either clinically or from CSF examination, in which case a neuropenetrative regimen should be used, such as procaine penicillin or a prolonged course of doxycycline, with careful follow-up. The use of intravenous penicillin G is also common in the treatment of neurosyphilis.

Because of the use of single dose macrolide antibiotics for other sexually transmitted infections, such as nonspecific urethritis and chlamydia, there is global macrolide resistance to syphilis so these antibiotics should not be used in the management of syphilis [13].

### Syphilis and HIV co-infection

The incidence of syphilis is increasing, particularly in HIV-positive patients. According to figures published by Public Health England, the number of reported cases of syphilis has reached the highest level in England since 1949 [14]. In Australia, the incidence of syphilis has been rising among men who have sex with men (MSM) since 2000 [15, 16].

Consequently, there has been an increasing number of cases of syphilis–HIV co-infection [17]. In Australia, the incidence of syphilis increased by 42% in HIV-negative men and 38% in HIV-positive men between 2010 and 2015 [18].

Whether syphilis and HIV transmission synergy is due to a biological phenomenon (i.e. mucosal ulceration), risk behaviour (i.e. a decrease in safer sex practices) or a combination of both, remains controversial.

The clinical manifestations of syphilis are almost identical in patients who are HIV-positive and HIV-negative. However, blurring of primary and secondary syphilis features has been described. Neurological invasion is more frequently seen in HIV-positive patients, with up to 70% having neurological invasion during early infection [17, 19–21]. This is more often asymptomatic but lumbar puncture is recommended in cases of suspected neurosyphilis. HIV and syphilis co-infected patients can also have delayed RPR/VDRL response to treatment, and historical studies have described a transient reduction in CD4+ cells and an increase in HIV viral load [17, 19–21].

Predictors of neurological syphilis in patients who are HIV-positive include headache, visual symptoms (e.g. blurry vision, vision loss, eye pain or red eye), low CD4+ count (not on antiretroviral therapy [ART]), high serum RPR/VDRL (> 1:32) and a detectable plasma viral load [22]. Visual symptoms may indicate ocular syphilis. Ocular syphilis tends to occur more frequently in patients who are HIV-positive, causing uveitis, retinitis, optic neuritis or retinal detachment [23].

The optimal treatment regimen for syphilis in patients who are HIV-positive is controversial and guideline recommendations in this population are based on limited data [24]. A neuropenetrative antibiotic regimen should be considered if the patient has neurological signs or symptoms, a low CD4+ count (< 350) in the absence of ART, high serum RPR/VDRL (> 1:32) and ocular disease [21].

Ultimately, efforts to prevent syphilis are needed. In a small randomised, controlled pilot study, Bolan et al. [25] demonstrated that prophylactic daily doxycycline reduced the incidence of syphilis among HIV-positive MSM who continue to engage in high-risk sex [25]. A larger follow-up study reported a 73% drop in syphilis infections in MSM who used doxycycline as on-demand post-exposure prophylaxis [26].

Nevertheless, prophylaxis is only one aspect of syphilis prevention. Effective prevention of syphilis also requires accurate surveillance, monitoring for treatment failure and resistance, diagnostic testing, early treatment, partner notification, the treatment and education of health workers and other at-risk populations.

### Eliminating HCV and HIV co-infection in Australia

HIV infection accelerates HCV disease progression in co-infected patients, and liver disease is a leading cause of non-AIDS-related mortality among patients who are HIV-positive [27]. To reduce the morbidity and mortality associated with HIV and HCV co-infection, all patients with HIV should be screened for HCV [28] and there should be universal access to HCV treatment [29].

The elimination of HCV in HIV co-infected patients in Australia requires continual intervention measures to reduce HCV incidence and HCV-related mortality [30]. Highly effective therapies, universal access to these therapies, a broader prescriber base, novel models of care, harm reduction, strategies to reduce reinfection, enhanced screening and diagnosis, careful and deliberate evaluation of results are key to the elimination of HCV in this patient community.

### Treatment of HCV in patients who are HIV-positive

Direct-acting antivirals (DAA) are used to treat HCV and the efficacy and tolerability of these therapies have improved over time. New HCV therapies provide similar sustained virological responses (SVR) in patients co-infected with HCV and HIV and patients infected with HCV alone [31–37].

There are new pan-genotypic regimens for treating patients co-infected with HCV and HIV. The ASTRAL-5 study reported a SVR of 95% for the sofosbuvir/velpatasvir (SOF/VEL) combination [38] and the EXPEDITION-1 study reported a SVR of 98% for the glecaprevir/pibrentasvir (GLE/PIB) combination [39].

HIV co-infection creates unique considerations for patients with HCV, particularly potential drug interactions between HCV DAAs and HIV ARTs (Table 2).

**Table 2:** Potential DAA/ART drug interactions

DAA	ART	Change required
DCV	EFV	Increase DCV dose to 90 mg
DCV	NVP/ETR	Likely increase DCV dose to 90 mg (no data)
DCV	ATZ + EVG/COBI/FTC/TDF	Decrease DCV dose to 30 mg
LDV/SOF	TDF-based regimens	Use with caution, esp. if renal impairment
PROD	ART	Many drug interactions—do not use
ELB/GRZ	PIs	Contraindicated—do not use
ELB/GRZ	NNRTIs (except RLV)	Contraindicated—do not use
ELB/GRZ	EVG/COBI/FTC/TDF	Contraindicated—do not use
SOF/VEL	EFV	Contraindicated—do not use

Data source: HepC Drug Interactions (http://www.hep-druginteractions.org)

*ATZ* atazanavir, *COBI* cobicistat, *DCV* daclatasvir, *ELB* elbasvir, *ETR* etravirine, *EFV* efavirenz, *EVG* elvitegravir, *FTC* emtricitabine, *GRZ* grazoprevir, *LDV* ledipasvir, *NNRTIs* non-nuclease reverse-transcriptase inhibitors, *NVP* nevirapine, *PIs* protease inhibitors, *PROD* paritaprevir/ritonavir–ombitasvir and dasabuvir, *RPV* rilpivarine, *SOF* sofosbuvir, *TDF* tenofovir disoproxil fumarate

Even with potent ART, co-infected patients are at increased risk of rapidly progressive liver disease. ART is not a substitute for HCV treatment. In Australia, several DAA regimens have been subsidised since March 2016, with no restrictions based on liver disease stage, drug or alcohol use. Between March 2016 and June 2017, an estimated 43,390 people living with HCV initiated DAA treatment (approximately 19% of the total HCV-positive population) [40].

### Impact of DAA regimens on HCV prevalence in HIV-positive patients in Australia

The Control and Elimination within AuStralia of HEpatitis C from people living with HIV (CEASE) observational cohort study aims to monitor progress towards elimination of HCV infection from the HIV-positive population [41]. In the first analysis, 390 HIV-positive patients with past or current HCV infection aged 18 and older were enrolled across 18 sites in Australia. The majority of the cohort was male (95%), gay or bisexual (84%), and on combination ART (94%) [41].

In the CEASE cohort, there was an 80% increase in cumulative HCV treatment after interferon-free DAA therapy became publically available, compared to two years prior [41]. SVR12 increased from 70% in 2014 to 92% in 2016, and HCV RNA prevalence decreased from 79% in 2014 to 28% in 2016 [41]. Among gay or bisexual males in the CEASE cohort, there was a significant inverse association between injecting drug use (IDU) in the last month and DAA uptake (odds ratio 0.51, 95% confidence interval 0.29–0.91) [41].

In addition to the use of effective therapies, elimination of HCV also requires harm reduction. In the CEASE cohort, there were high levels of pre-treatment risk behaviour—81% reported IDU ever, 31% reported IDU in the past 6 months and 25% reported IDU in the past month. Of MSM who engaged in casual sex in the past 6 months, 13% never disclosed their HIV status and 44% never disclosed their HCV status [42].

The Australian Trial in Acute Hepatitis C (ATAHC) study previously identified clusters of HCV strains in HIV-positive patients who acquired HCV through IDU and sex, irrespective of the mode of infection [43]. Understanding transmission networks may also be key to eliminating HCV.

## Proctitis and antimicrobial resistance in the HIV clinic

Sexually transmitted infections causing proctitis occur in MSM and therefore proctitis can be seen in the HIV clinic. *Chlamydia trachomatis* (including lympho-granuloma venereum), *N. gonorrhoeae*, syphilis, herpes simplex virus, and possibly *M. genitalium* can all cause sexually-acquired proctitis.

Treatment of suspected sexually-acquired proctitis should be commenced prior to test results being available. The Australian Sexual Health Alliance STI Management Guidelines recommend immediate treatment of proctitis using ceftriaxone, doxycycline and valacyclovir [44]. This is because it can be very difficult to distinguish between anorectal gonorrhoea, chlamydia and herpes simplex infection on clinical grounds.

### *N. gonorrhoeae* antimicrobial resistance

With the increasing incidence of gonorrhoea in the wider community, *N. gonorrhoeae* antibiotic resistance is an emerging issue in the HIV clinic [18]. The current treatment recommendation for gonorrhoea is a stat dose of 500 mg ceftriaxone administered via IMI with lignocaine, along with 1 g azithromycin administered orally. Other antimicrobials used to treat *N. gonorrhoeae*, including ciprofloxacin, doxycycline and gentamicin, if test results demonstrate susceptibility.

Increasing rates of *N. gonorrhoeae* resistance to these antimicrobials have been reported [45]. There are numerous reports of ceftriaxone failing to treat pharyngeal gonorrhoea cases [46–50], and there has been one reported case of ceftriaxone and azithromycin failing to treat a *N. gonorrhoeae* infection [51]. Due to extremely high levels of antimicrobial resistance, cefixime is no longer recommended as a treatment for gonorrhoea [52, 53].

Recently, there have been cases of high-level resistance to azithromycin in the UK [54], Hawaii [55] and South Australia [56]. Notably, all 50 cases with azithromycin-resistant gonorrhoea in South Australia were susceptible to ceftriaxone [56].

As gonorrhoea is fast becoming the next ‘super-bug’, it is extremely important to culture gonorrhoea whenever it is treated to obtain data regarding antibiotic sensitivities [44].

### *M. genitalium* antimicrobial resistance

More studies need to be conducted to determine if *M. genitalium* causes proctitis. A study at the Melbourne Sexual Health Centre identified *M. genitalium* in 21% of patients with proctitis who were HIV-positive and 8% of patients with proctitis who were HIV-negative. The *M. genitalium* bacterial load was found to be six times higher among rectal infections with proctitis symptoms compared to asymptomatic *M. genitalium* infection [57]. Studies examining an association with symptoms and anorectal *M. genitalium* detection give conflicting results [58, 59]. Evidence that *M. genitalium* causes urethritis is much stronger [60].

Treating *M. genitalium* has become a major concern due to its resistance profile [61]. *M. genitalium* lacks a cell wall and has few antibiotic targets. Azithromycin treatment failure in wild-type infections selects mutations in 23S rRNA (macrolide resistance mutations [MRM]) [62, 63]. MRM have been found to be present in more than 80% of *M. genitalium* infections detected at the Melbourne Sexual Health Centre and MRM is the strongest predictor of azithromycin treatment failure, although bacterial load also appears to be important.

Though various antibiotic regimes have been used, the ideal treatment for *M. genitalium* infection is not known. Extended azithromycin (1.5 g over 5 days) is not more effective than a single 1 g dose at achieving cure of *M. genitalium* urethritis [63]. Moxifloxacin can be used as a second-line treatment, but fluoroquinolone resistance is increasing. In Japan, fluoroquinolone resistance incidence is 47% [64] and in Australia, fluoroquinolone resistance is 14%, with a combined macrolide–fluoroquinolone resistance of 9% [65]. The third-line treatment, pristinamycin with or without doxycycline, cures about 75% of infections [66].

Given the likelihood that *M. genitalium* will be resistant to macrolides in cases of proctitis, treatment of *M. genitalium* remains a dilemma. PCR can detect resistant *M. genitalium* to inform treatment based on the presence of MRM; however, this test is not available at all centres. Because macrolide resistance is so common in MSM, it is reasonable to assume it is present in this group. There are few options for treating *M. genitalium* other than macrolides and moxifloxacin, so screening for this organism is not recommended. However, treatment is required for patients with symptoms attributable to the infection, and for partners in an ongoing sexual relationship as rectal infection may be present in over 40% of male partners of infected men [67].

## Conclusions

Efforts to prevent, treat and reduce ongoing transmission and incidence of STIs in patients with HIV are underway, but further advances are needed to reduce the significant morbidity associated with these common infections. Preventing co-infection of STIs in HIV-positive patients may be possible through increased testing and prudent management, reduced risk-behaviour and ultimately, by elimination of the microbe. Management of patients who are already co-infected should be based on the increasing body of evidence pertaining to drug–drug interactions and antimicrobial resistance.

## Abbreviations

ART: antiretroviral therapy
CEASE: Control and Elimination within AuStralia of HEpatitis C from people living with HIV
COBI: cobicistat
CSF: cerebrospinal fluid
DAA: direct-acting antivirals
DCV: daclatasvir
EFV: efavirenz
EIA: enzyme immunoassay
ELB: elbasvir
EVG: elvitegravir
FTC: emtricitabine
GLE: glecaprevir
GRZ: grazoprevir
HCV: hepatitis C virus
IDU: injecting drug use
IgG: immunoglobulin G
IgM: immunoglobulin M
IMI: intramuscular injection
LDV: ledipasvir
MIC: minimum inhibitory concentration
MRM: macrolide resistance mutations
MSM: men who have sex with men
NNRTIs: non-nuclease reverse-transcriptase inhibitors
PCR: polymerase chain reaction
PIB: pibrentasvir
PIs: protease inhibitors
PrEP: pre-exposure prophylaxis
PROD: paritaprevir/ritonavir–ombitasvir and dasabuvir
RPR: rapid plasma reagin
RPV: rilpivarine
SOF: sofosbuvir
STIs: sexually transmitted infections
SVR: sustained virological response
TDF: tenofovir
TPHA: *Treponema pallidum* hemagglutination assay
TPPA: *Treponema pallidum* particle agglutination assay
VDRL: venereal disease research laboratory
VEL: velpatasvir
